# Lean Six Sigma for Sharps Waste Management and Occupational Biosafety in Emergency Care Units

**DOI:** 10.3390/ijerph23010122

**Published:** 2026-01-19

**Authors:** Marcos Aurélio Cavalcante Ayres, Andre Luis Korzenowski, Fernando Elemar Vicente dos Anjos, Taisson Toigo, Márcia Helena Borges Notarjacomo

**Affiliations:** 1 Department of Production Engineering, Unisinos—Universidade do Vale do Rio dos Sinos, São Leopoldo 93022-750, Brazil; marcos.a@unitins.br (M.A.C.A.); akorzenowski@unisinos.br (A.L.K.); 2 Instituto Federal de Educação, Ciência e Tecnologia do Rio Grande do Sul (IFRS), Campus Caxias do Sul, Caxias do Sul 95043-700, Brazil; taisson.toigo@caxias.ifrs.edu.br; 3 Departmentof Production Engineering, Universidade Estadual do Mato Grosso do Sul—UEMS, Dourados 79804-970, Brazil; notarjacomo@hotmail.com

**Keywords:** lean six sigma, DMAIC, healthcare waste management, sharps waste, occupational health, biosafety, public health governance, environmental sustainability, continuous improvement, health services management

## Abstract

**Highlights:**

**What are the main findings?**

- Needlestick injuries represent a critical occupational risk in Emergency Care Units, with direct implications for healthcare workers’ safety and biosafety governance.
- The application of Lean Six Sigma (DMAIC) is significant in structuring low-cost managerial interventions aimed at process standardization, traceability, and risk control in public healthcare services.

**What are the implications of the main findings?**

- The proposed improvement plan offers a replicable approach to strengthening biosafety governance and reducing occupational risks in resource-limited emergency and urgent care settings.

**Abstract:**

Occupational exposure to sharps waste represents a critical challenge for public health systems, directly affecting healthcare workers’ safety, institutional costs, and environmental sustainability. This study aimed to analyze sharps waste management practices and to structure improvement actions for biosafety governance in Brazilian Emergency Care Units (ECUs) through the application of the Lean Six Sigma (LSS) and DMAIC method (Define, Measure, Analyze, Improve, and Control). A single multiple-case study was conducted across three public units in different regions of Brazil, combining direct observation, regulatory checklists based on ANVISA Resolution No. 222/2018 (RDC), and cause–and–effect (5M) analysis. The diagnostic phase identified recurrent nonconformities in labeling, documentation, and internal transport routes, primarily due to managerial and behavioral gaps. Based on these findings, the DMAIC framework supported the development of a low-cost, evidence-based action plan that outlined proposed interventions, including visual checklists, standardized internal routes, and key performance indicators (KPIs), intended to strengthen biosafety traceability and occupational safety. The se proposed actions are expected to support continuous learning, staff engagement, and a culture of shared responsibility for safe practices. Overall, the study provides a structured basis for future implementation and empirical validation of continuous improvement initiatives, aimed at enhancing public health governance and occupational safety in resource-constrained healthcare environments.

## 1. Introduction

Occupational hazards affecting healthcare professionals represent a major biosafety challenge in hospital environments, with direct impacts on occupational health, institutional costs, and the continuity of healthcare services. Among these accidents, those caused by sharp materials—such as needles, blades, and catheters—are among the most frequent sources of exposure to biological and chemical agents, requiring preventive policies, strict protocols, and safe handling practices. Accidental exposure to body fluids through sharp injuries is associated with a high risk of pathogen transmission, including hepatitis B, hepatitis C, and HIV, making the proper management of such waste a priority for the safety of healthcare workers and for the integrity of public health services [1,2,3,4], and when failures in segregation and traceability occur, sharps waste may enter municipal waste streams and landfills, posing serious public and environmental health risks [5,6,7].

In the Brazilian context, sharps waste is classified as Group E under Resolution RDC No. 222/2018 (RDC), issued by the National Health Surveillance Agency [8,9] (ANVISA), which regulates the management of healthcare waste. This regulation establishes requirements for infrastructure, internal flow, segregation, identification, temporary storage, and traceability of documentation within the framework of the Healthcare Waste Management Plan (PGRSS). However, in practice, many ECUs — Brazilian public 24/7 urgent care units within the Unified Health System (SUS) dedicated to urgent and emergency care—face resource limitations and management gaps that hinder full compliance with RDC requirements. Among the most recurrent problems are labeling errors on containers, the absence of standardized internal transport routes, and delays in filing certificates of final waste disposal [1].

To support the analysis and structuring of improvement actions, the LSS has proven a practical approach by combining waste elimination with control of operational variability. Its DMAIC [10] offers a systematic structure for defining problems, measuring performance, analyzing root causes, planning improvement actions, and consolidating routine controls [11,12]. In sharps waste management, DMAIC can support standardization, measurement, and sustainable, safe practices, with a low implementation cost and a focus on measurable results.

This research is justified, first, by the social and sanitary relevance of the theme [13,14]. Strengthening the management of sharps waste in ECUs has the potential to reduce occupational risks and biological exposure, contributing to safer environments for both healthcare professionals and patients. Well-structured and controlled processes are expected to support worker motivation and confidence, reduce fear related to exposure to infectious materials, and foster engagement with biosafety practices [13]. Such conditions may contribute to lower absenteeism, fewer work-related absences, and reduced staff turnover, while promoting the retention of qualified professionals, thereby positively impacting the continuity and quality of public health services [15].

In addition to social and occupational dimensions, the study also presents relevant economic implications. More standardized and supervised routines are expected to reduce material waste, rework, and segregation errors, thereby lowering operational costs in public health institutions. The rationalization of collection, transport, and documentation processes may allow better use of human and material resources, supporting the financial sustainability of urgent and emergency care services [16,17,18,19].

From a scientific perspective, the rationale for this study lies in recognizing that much of the literature emphasizes structural, physical, or technological solutions—such as equipment acquisition or infrastructure upgrades—while there remains a lack of studies addressing managerial capacity, continuous improvement culture, and the consolidation of low-cost verification routines [20,21,22]. This study proposes the use of DMAIC as an analytical and planning tool in public health environments, highlighting that leadership capacity and systematic routines are frequently identified in the literature as determinants of sustainable performance. Therefore, the study contributes conceptually to discussions on operational efficiency, biosafety governance, and sustainability within SUS.

Beyond the regulatory dimension, the literature highlights that safe practices and consistent waste-management routines also depend on organizational factors, including safety culture, continuous learning, and managerial capacity. Safety culture directly influences risk perception, adherence to procedures, and commitment to safe behaviors [23,24]. Likewise, organizational learning—supported by feedback cycles, indicator review, and the consolidation of standards—contributes to more stable verification routines and to the document-governance practices required for traceability [21,25]. However, studies show that these managerial dimensions remain underexplored in healthcare services, particularly in emergency units, where care pressures often privilege structural or technological solutions over low-cost mechanisms for continuous improvement [22].

Beyond its technical-operational nature, the proposed approach also involves human and educational dimensions that are essential for sustaining improvements if implemented in future contexts. The application of DMAIC in public health settings requires, in principle, the active participation of professionals and managers to encourage safe behaviors and shared responsibility. The planned development of visual routines, short training sessions, and continuous feedback may function as educational and psychological mechanisms, reinforcing organizational learning and the internalization of safe practices. In this way, the study is positioned at the intersection of health, organizational psychology, and workplace education, contributing conceptually to competence development and biosafety culture.

Based on this context, this study addresses the following research question: How can DMAIC be used to assess ECUs’ compliance with RDC, identify the managerial, behavioral, and operational causes of the main non-conformities, and structure low-cost improvement actions that strengthen biosafety and governance in sharps waste management processes? This research adopts a multiple-case study design; therefore, the findings are not statistically generalizable.

Thus, this study aims to analyze the internal flow of sharps waste management in ECUs, using the DMAIC framework to identify failures, structure proposed improvements, and develop a low-cost action plan, without reporting the implementation or empirical evaluation of the proposed actions.

## 2. Materials and Methods

### 2.1. LSS and the DMAIC Method

LSS is a continuous improvement methodology widely applied in industrial contexts and, more recently, in healthcare services [10]. Its purpose is to reduce waste, control variability, and improve process efficiency. LSS combines the principles of Lean Manufacturing, focused on removing non-value-adding activities, with the statistical techniques of Six Sigma, aimed at controlling variability and supporting performance quality management [26].

The DMAIC structure for LSS application divides the LSS application into five sequential, interdependent stages. In the Define phase, the problem and critical-to-quality parameters (CTQs) are established; in Measure, data and performance indicators are collected; in Analyze, approaches are applied to prioritize the most critical root causes from a biosafety and regulatory compliance perspective. This prioritization supports the structured definition of improvement actions to be proposed in the subsequent DMAIC phase (Improve), ensuring that the planned interventions are proportional to the identified risks’ impact and urgency. Finally, in the Control phase, mechanisms for monitoring process performance are defined, aiming to support future follow-up activities [10].

When applied to healthcare services, DMAIC can support the standardization of clinical and administrative processes, reduce errors and costs, and strengthen occupational and patient safety. In the context of this study, the method was adopted as an analytical and planning framework to map and analyze the flow of sharps waste management in Emergency Care Units (ECUs), providing structured technical and managerial inputs to support decision-making, without reporting the implementation of improvement actions [27,28].

### 2.2. Regulatory Framework: RDC and the Healthcare Waste Management Plan (PGRSS)

The RDC issued by the National Health Surveillance Agency [8] establishes the guidelines for healthcare waste management (RSS) in Brazil. It defines criteria for the segregation, identification, packaging, storage, transport, treatment, and final disposal of waste, setting minimum biosafety and traceability standards.

Sharps waste, classified as Group E, requires special care due to the risk of accidents and occupational contamination. The regulation stipulates that these materials must be disposed of in rigid, leak-proof, puncture-resistant, and properly labeled containers, as part of the institution’s Healthcare Waste Management Plan (PGRSS).

In this study, the RDC is adopted as a regulatory reference and analytical compliance benchmark, enabling the DMAIC framework to assess adherence to legal requirements and to structure proposed process improvement actions aimed at strengthening biosafety governance and operational control, without reporting the implementation or empirical evaluation of such actions in the analyzed ECUs.

### 2.3. Study Design and Methodological Procedures

This study adopts a single multiple-case study design, conducted across three ECUs within the Brazilian public healthcare system, located in the municipalities of São José (Imperatriz–MA), Augustinópolis (TO), and Bom Jardim (Parauapebas–PA) (MA (Maranhão), TO (Tocantins) and PA (Pará) refer to the Brazilian states in which the Emergency Care Units analyzed in this study are located: São José (Imperatriz–MA), Augustinópolis (TO), and Jardim (Parauapebas–PA)). The selection of the three Emergency Care Units followed an intentional exploratory multiple-case design, consistent with the logic of analytical rather than statistical generalization. The units were purposely chosen to capture contextual variability across different states and operational environments within SUS. The geographical distances between the ECUs—approximately 140 km between Imperatriz–MA and Augustinópolis–TO, 360–380 km between Augustinópolis–TO and Parauapebas–PA, and about 500 km between Imperatriz–MA and Parauapebas–PA—reinforce the territorial heterogeneity included in the study. This approach follows the established guidance for a single multiple-case methodology [29,30,31].

Data collection was conducted through on-site observation by researchers, including direct monitoring of segregation, internal transport, storage, and documentation of Group E waste [10,32]. These observations supported the development of a cause-and-effect matrix (5M), structured into the categories Method, Manpower, Material, Measurement, and Milieu (Environment), to identify the most frequent causes of non-conformity and the critical failure points within the process. The data used to construct the 5M Cause–Effect Matrix (Table 1) were obtained from three integrated sources: (1) direct observation of segregation, internal transport, and storage practices; (2) systematic non-conformity records derived from the RDC checklist (Appendix A, Table A1); and (3) structured field verification and brief interactions with frontline staff, cleaning teams, and unit coordinators. These sources were triangulated to classify empirical evidence into the five 5M categories (Method, Manpower, Material, Measurement, and Milieu).

Subsequently, a checklist based on RDC was applied, covering the domains of infrastructure, process flow, and documentation governance, to assess each unit’s compliance with regulatory and biosafety requirements. During the analysis stage, the collected data were organized and evaluated using the DMAIC framework.

To ensure methodological reliability, data collection and checklist scoring were performed by the same trained researcher across all three ECUs, ensuring procedural consistency and minimizing inter-observer variability. The checklist was also pre-tested in non-sampled ECUs before data collection, allowing refinement of item wording and observation procedures to provide clarity and operational consistency across sites.

- In the Define phase, the study scope, sample, and critical-to-quality parameters (CTQs) to be observed were established.
- In Measure, systematic data collection was carried out through observations and checklists.
- In the Analyze phase, a cause-and-effect matrix was developed, and the GUT (Gravity, Urgency, and Trend) prioritization method was applied to rank the identified problems and guide improvement efforts.
- The Improve phase consisted of proposing a continuous improvement plan, including low-cost practices such as the reorganization of internal flows, visual routines, labeling standardization, and operational training.
- Finally, in Control, performance indicators (KPIs) and control points were defined for periodic monitoring, ensuring the maintenance of the achieved results.

The data and findings obtained in the previous stages were systematized and will subsequently be presented as results, followed by the discussion and final considerations, which address the practical and managerial implications of applying the DMAIC to sharps waste management in public ECUs.

## 3. Results

The results of this study were organized according to the five phases of the DMAIC, which guided both data collection and interpretation. In the Define phase, the initial findings indicated similar patterns across the three units, with partial compliance in infrastructure but significant weaknesses in labeling, documentation, and internal route standardization. This convergence suggests that the causes of non-conformity are not solely material constraints but also managerial and behavioral factors—particularly the absence of systematic training and limitations in direct supervision.

Table 1 presents the Cause–Effect Matrix (5M), which summarizes the leading observed causes and their relationships with the critical-to-quality (CTQ) parameters defined for sharps waste management in Emergency Care Units. The empirical evidence supporting the construction of the 5M matrix results from systematic qualitative field observations conducted across the three Emergency Care Units analyzed, consolidated through direct on-site observation, document verification, and the application of regulatory checklists. For methodological transparency and qualitative data traceability, the detailed field observations by unit are presented in Appendix B, Table A2. Although no recent accidents were recorded in the analyzed units (over the past 5 years), field observations revealed conditions that increase the potential for occupational exposure, mainly due to the lack of standardized routines and weak supervision mechanisms.

After developing the 5M matrix, the identified causes were evaluated and prioritized using the GUT method (Gravity, Urgency, and Trend) to determine which failures posed the most significant immediate safety and compliance risk. The most severe causes and the highest recurrence trend were associated with the lack of standardized routines, the absence of systematic training, and gaps in final disposal documentation. This prioritization allowed improvement efforts to focus on the most impactful areas, serving as the foundation for the action plan presented in the subsequent sections. Table 2 summarizes the GUT scores used to prioritize the causes identified in the 5M matrix, and the site-level GUT scores assigned to each Emergency Care Unit that support the consolidated prioritization are presented in Appendix C, Table A3.

The highest priority was assigned to the absence of standardized verification routines (score 14), followed by the lack of systematic and documented staff training (score 13), and incomplete documentation of final disposal certificates (score 12). Additional causes, such as inadequate labeling and biosafety pictograms and the absence of monitoring indicators, received intermediate scores and were also considered within the analytical scope of the Improve phase of the DMAIC. This prioritization supported the structured definition and sequencing of proposed improvement actions outlined in the Continuous Improvement Plan.

The consolidated analysis of the three ECUs revealed convergent patterns of nonconformity, primarily within the Method, Manpower, and Document Governance categories, supporting the interpretation that the observed deficiencies stem less from a lack of physical resources than from gaps in management and continuous monitoring.

The Method axis showed the absence of formalized verification routines, of internal collection schedules, and of defined criteria for container replacement. Such conditions are associated with increased operational variability and potential overflow risk, which may compromise biosafety and the traceability required by RDC.

In the Manpower axis, a lack of systematic training and the absence of training records were identified, which may contribute to inconsistencies in segregation, container handling, and the use of personal protective equipment (PPE). This training gap helps explain some of the recurring non-conformities and underscores the relevance of the capacity-building routines proposed in the continuous improvement plan.

Regarding the Material axis, irregular use of labels and pictograms was observed, with potential implications for legal compliance and professionals’ perception of safety. In the Measurement axis, the absence of indicators limits the ability to monitor performance and support organizational learning, while in the Environment axis, incomplete documentation of final disposal poses a potential legal and environmental risk.

Overall, the results indicate that the definition of standardized procedures, the establishment of performance indicators, and the strengthening of managerial capacity are frequently highlighted in the literature as key factors for compliance and safety in sharps waste management processes in public Emergency Care Units. These findings provide the analytical basis for the proposed continuous improvement plan and control routines, which are presented conceptually in the following sections.

### 3.1. Compliance Assessment According to RDC

In Brazil, the RDC, issued by the National Health Surveillance Agency (ANVISA), establishes guidelines for healthcare waste management and classifies sharps waste as Group E. The regulation requires that such materials be disposed of in rigid, leak-proof, puncture-resistant, and properly labeled containers, establishing requirements for documentary traceability within each institution’s Healthcare Waste Management Plan (PGRSS).

However, national evidence has repeatedly documented failures in labeling and the use of biosafety pictograms, as well as inconsistencies in the archiving of final disposal records, reinforcing the need for governance and control mechanisms aligned with regulatory requirements. In the present study, these requirements were operationalized through a checklist structured into three domains—infrastructure, process/flow, and governance/documentation—with dichotomous scoring (compliant/non-compliant), to preserve the traceability of critical-to-quality parameters (CTQs) throughout the Define, Measure, and Analyze phases of the DMAIC. The same standardized checklist was applied for diagnostic purposes across all three Emergency Care Units, allowing direct comparison of compliance levels. Table 3 summarizes the consolidated RDC compliance assessment and site-level diagnostic results, while the checklist instrument is provided in Appendix A, and the site-level operational evidence supporting the consolidated RDC compliance classification is presented in Appendix D, Table A4.

The application of the standardized checklist revealed heterogeneous levels of compliance among the three ECUs, with better performance in physical infrastructure—particularly in the availability of suitable rigid containers—but persistent weaknesses in processes and governance.

Inadequate container labeling, the absence of biosafety pictograms, and the lack of regular training were identified as the most critical non-conformities, directly affecting occupational safety and the traceability required by legislation. In addition, partial standardization of internal routes and inconsistencies in certificate archiving compromised managerial control and the transparency of disposal practices.

Another issue identified was the complete outsourcing of waste collection and disposal to municipal administrations. This contractual structure prevents ECU managers from having direct access to final disposal certificates or visibility over the external transport and treatment process. This lack of traceability constitutes a significant governance limitation, contradicting the principle of complete control established in the PGRSS and hindering full compliance with RDC. These results reinforce that compliance depends not only on structural adequacy but also on the implementation of management, training, and systematic verification mechanisms—elements that form the foundation of the continuous improvement plan developed in the following stages of this study.

### 3.2. Performance Indicators (KPIs) and Continuous Improvement Plan

In this study, the control plan was structured to promote regular management routines, systematic monitoring of performance indicators (KPIs), and clear accountability for processes, thereby reducing operational variability and strengthening the biosafety culture in ECUs. The Continuous Improvement Plan was developed by combining the causes prioritized through the GUT method with recommendations from the literature on Lean Healthcare, biosafety, and the implementation of improvement in healthcare settings. The deadlines were set as realistic estimates based on the complexity of each action and the three complementary criteria. Regarding complexity, low-effort, low-cost actions—such as standardization procedures, visual communication improvements, and verification routines—were classified as short-term (up to 60 days). Actions requiring staff training, workflow adjustments, or documentation integration were categorized as medium-term (60–120 days). More complex actions involving administrative coordination or external contracting were classified as long-term (more than 120 days). About three complementary criteria: (i) the regulatory criticality of each non-compliance according to ANVISA requirements, prioritizing actions directly related to occupational biosafety and legal conformity; (ii) the operational complexity and feasibility of the proposed actions within the context of Emergency Care Units; and (iii) the logic of the DMAIC improvement cycle, in which simpler, low-complexity interventions were assigned shorter timeframes, while actions involving training, behavioral change, or digital traceability were allocated longer planning horizons. These deadlines therefore represent expected implementation horizons rather than empirical results. This structure ensured operational feasibility and alignment with the ECUs’ managerial capacity. The improvement plan is presented in Table 4.

Implementing the proposed control plan institutionalizes continuous improvement within the ECUs’ operational routines, transforming occasional training and verification activities into permanent management practices. Field inspections (gemba walks) and semiannual audits strengthen managerial feedback, while the systematic review of indicators promotes transparency and organizational learning.

In addition, the combination of periodic training sessions and feedback meetings helps consolidate a culture of shared responsibility, in which managers and frontline professionals recognize the impact of their actions on occupational and environmental safety.

The literature emphasizes that, in public and resource-constrained environments, low-cost control mechanisms and local managerial ownership—such as visual inspections, checklists, and KPI monitoring—are essential to sustaining compliance and reducing risks [10,11,23,33,34]. Thus, the control plan proposed here represents not merely the final stage of the DMAIC but a mechanism of continuous governance, capable of ensuring the stability of results and fostering managerial maturity within the analyzed units [33,34,35,36].

At the end of the methodological cycle, the performance indicators constitute the Control phase of the DMAIC, ensuring the sustainability and feedback of the implemented improvements. These indicators enable continuous process monitoring, ensuring that corrective actions go beyond one-time interventions and become permanent management practices.

Systematic monitoring of indicators also enables trend analysis, deviation detection, and feedback to local managers, thereby strengthening decision-making capacity and the institutional culture of biosafety. In this way, the indicators play a central role in governance and in the standardization of sharps waste management processes, translating the critical-to-quality parameters established in earlier stages of the study into measurable operational metrics.

The indicators included in the Control Phase were selected based on three complementary criteria. First, each indicator reflects a Critical-to-Quality (CTQ) requirement identified during the Define, Measure, and Analyze phases of the DMAIC, focusing on elements that most directly affect biosafety and compliance. Second, the indicators incorporate the RDC’s regulatory requirements, particularly those related to segregation practices, container identification, visual conformity, storage conditions, and the traceability of documentation. Third, the selection was guided by evidence from recent studies showing that simple, low-cost compliance indicators—such as visual inspections, checklist adherence, and routine monitoring—enhance process reliability and strengthen local safety governance in resource-constrained healthcare settings [33,34,36]. Based on these criteria, the final set of indicators proposed in Table 5 aims to ensure operational feasibility, strengthen biosafety practices, and support continuous managerial oversight.

The following presents the group of indicators proposed for the sharps waste management process in Emergency Care Units, as shown in Table 5.

The indicators defined in Table 5 align with the Control phase of the DMAIC, enabling the transformation of improvement actions into measurable, sustainable practices. Each KPI translates a critical-to-quality (CTQ) parameter into an objective metric, allowing continuous monitoring of compliance with RDC and biosafety principles. The definition of these indicators was informed by established recommendations in the Lean Healthcare, occupational safety, and healthcare waste management literature, which emphasize visual management, incident tracking, and process stability as key elements of biosafety governance, e.g., [6,10,11,23,33]. These indicators—such as correct container labeling, sharps incident rate, average internal flow time, and timely archiving of certificates—enable evidence-based management, strengthening local governance and process transparency. By providing regular, comparable data, KPIs allow the detection of deviations, inform managerial decision-making, and prevent performance decline. In addition, their operational simplicity and low cost facilitate their incorporation into the routine operations of Emergency Care Units, ensuring continuity and organizational learning. Thus, the indicators function as the link between continuous improvement and the sustainability of biosafety practices within the scope of SUS, and from a methodological perspective, these KPIs are intended to be monitored through routine document verification, visual inspections, checklist-based assessments, and occupational safety records, ensuring feasibility and consistency within the operational context of ECUs.

## 4. Discussion

The results of this study underscore the importance of systematic sharps waste management in ECUs, highlighting that the observed deficiencies stem less from a lack of structural resources than from gaps in management, monitoring, and training [2,3,4]. Beyond structural and procedural factors, the findings suggest that the consolidation of improvements is likely to depend heavily on behavioral and motivational factors. The proposed use of checklists, feedback mechanisms, and recurring training sessions is expected to strengthen process control and may enhance professionals’ sense of self-management and ownership, based on patterns identified during the diagnostic phase and evidence reported in the literature [10,11,23,33,34]. This perspective aligns with contemporary models of organizational learning, which emphasize practical involvement and positive reinforcement in the internalization of safe habits. Recent evidence indicates that periodic training sessions, combined with structured feedback mechanisms, are associated with organizational learning, improve adherence to biosafety procedures, and consolidate a culture of shared responsibility between managers and frontline staff. Continuous education cycles reinforce correct practices, while feedback meetings promote bidirectional communication, enhance accountability, and sustain engagement with safety routines in resource-constrained healthcare environments [34,36,37]. These findings support the premise adopted in this study that training and feedback function as behavioral reinforcement mechanisms essential to maintaining compliance with sharps waste management standards. The DMAIC is positioned as a facilitator of social and psychological learning, aligning biosafety practices with the development of meaning and belonging within healthcare teams.

These findings align closely with theoretical frameworks related to safety culture in healthcare, which argue that operational reliability depends not only on formal protocols but also on the internalization of safe practices, shared risk perception, and active supervisory engagement [23,24]. The variability observed in ECUs routines can be interpreted through the lens of organizational learning: organizations with low learning maturity tend to exhibit recurrent gaps in standardization, limited feedback cycles, and difficulty consolidating continuous improvement practices [21,25]. Furthermore, change-management models indicate that the incorporation of new behaviors—such as verification routines, systematic recording, and structured checks—requires consistent reinforcement mechanisms, including periodic training, structured feedback, and leadership capable of sustaining change over time [38]. Together, these theoretical perspectives help explain why managerial and behavioral interventions are as critical as structural or technological solutions in preventing non-conformities in sharps waste management.

In addition to operational and biosafety improvements, the potential economic implications also warrant consideration. Although the improvement plan was not empirically implemented, the diagnostic findings allow prospective identification of possible financial benefits, reduced variability, standardized routines, and stronger documentation governance. The literature indicates that failures in segregation practices, inconsistencies in internal routes, and delays in certificate archiving lead to material waste, rework, increased operational costs, and exposure to regulatory penalties [9,15,16,19]. Therefore, as a prospective analysis, the results suggest that applying DMAIC may support more efficient use of human and material resources, minimize operational losses, and enhance the financial sustainability of emergency care units, particularly in resource-constrained environments. These expected economic gains reinforce the managerial relevance of the proposed approach and justify future studies involving practical implementation and longitudinal cost assessment. In addition, improved sharps waste management also generates relevant environmental benefits, as standardized segregation, labeling, and traceability reduce the risk of improper disposal, environmental contamination, and regulatory non-compliance, contributing to safer waste treatment and greater environmental sustainability in public healthcare services [9,15,16,19].

Table 1, which summarizes the Cause–Effect Matrix (5M) [32], revealed that the main factors associated with non-conformities are concentrated in the Method, Manpower, and Document Governance categories. The absence of standardized verification routines, empirical criteria for container replacement, and labeling failures were the most recurrent critical points. These findings align with previous studies showing that, in hospital environments in developing countries, the most critical shortcomings are associated with low managerial maturity and the absence of a continuous improvement culture [1,16,19,20,22]. Similarly, Ozder et al. (2013) [14] and Kumar et al. (2015) [13] observed that irregular training and lack of regulatory knowledge are factors directly associated with the recurrence of unsafe practices in sharps waste handling. Thus, the results obtained here reinforce the literature by demonstrating that the predominant causes of non-conformity are not related to material availability but instead to the absence of leadership, monitoring, and continuous organizational learning.

Table 2, which refers to compliance with RDC, showed heterogeneous levels of adherence among the analyzed units [5], with satisfactory performance in infrastructure but marked deficiencies in the process and governance domains. Irregular labeling, lack of biosafety pictograms, and the absence of systematic training records were identified as the most critical non-conformities. These results corroborate the findings of Ferreira et al. (2020) [9] weakening of biosafety. Furthermore, the lack of access to final disposal certificates, resulting from the outsourcing of waste collection services, constitutes a structural governance limitation previously noted by Delevati et al. (2019) [1] and Alighardashi et al. (2024) [27], who emphasize that the direct accountability of generating units is an indispensable condition for effective compliance with the regulation. Thus, the present study confirms that regulatory compliance requires not only adequate infrastructure but also internal management and supervisory mechanisms that ensure traceability and local accountability.

Table 3, which presents the Continuous Improvement Plan, illustrates how the DMAIC method can guide the development of low-cost, high-impact interventions to address the leading causes of operational variability; however, these actions were not implemented, and therefore their effects are discussed as expected outcomes. The adoption of tools such as Kaizen Blitz, Kanban, Value Stream Mapping, and SIPOC strengthens standardization, visual communication, and collective learning. These strategies have been widely recognized in the Lean Healthcare literature as effective for reducing waste, enhancing safety, and consolidating sustainable practices in public healthcare services [10,11,12]. In line with Fletcher et al. (2021) [28], integrating DMAIC with visual management tools has strong potential to improve waste traceability and continuously reduce occupational risks. Furthermore, the direct involvement of operational teams in short improvement cycles fosters a sense of ownership and shared responsibility [18] as essential for behavioral change and the strengthening of a biosafety culture.

Table 4, which consolidates the KPI Dictionary from the Control phase, represents a significant advancement in operationalizing the DMAIC in public healthcare environments. The proposed indicators—such as the correct labeling rate, sharps incident rate, average internal flow time, and percentage of certificates archived on time—translate the critical-to-quality parameters (CTQs) into objective and easily monitored metrics. The implementation of these KPIs lays the foundations for evidence-based governance, enabling the detection of deviations, feedback on decision-making processes, and the long-term sustainability of results. This approach aligns with the principles of continuous improvement in LSS [10,26] and reinforces the understanding that performance measurement is a key pillar of organizational maturity. Furthermore, recent literature indicates that, in resource-constrained contexts, the adoption of simple, accessible, and periodically reviewed indicators is a decisive factor in sustaining improvements [17,27,33,35].

In addition to operational and regulatory impacts, the improvements proposed in this study have direct effects on organizational climate and the motivation of healthcare professionals. The creation of safer and more predictable environments reduces the fear of accidents, increases engagement, and strengthens confidence in institutional practices, as noted by Kumar et al. (2015) [13] and Conti et al. (2024) [18]. The perception of workplace safety is strongly associated with job satisfaction and individual performance, leading to lower turnover, greater adherence to routines, and more substantial collective commitment to biosafety. The strengthening of local leadership and the institutionalization of feedback and learning routines also foster team self-confidence, transforming waste management from a bureaucratic obligation into a driver of positive organizational culture.

Taken together, the four tables demonstrate the applicability and relevance of DMAIC as a management and training tool in public healthcare settings. The model proposed in this study achieved gains in standardization, traceability, safety, and motivation without requiring significant infrastructure investments—confirming the feasibility of low-cost, high-return managerial solutions. Beyond operational benefits, the findings reveal progress in understanding biosafety as the outcome of consistent managerial practices and a healthy psychosocial environment, rather than merely as a result of structural adjustments.

In terms of contributions, this study expands the literature by empirically demonstrating that LSS, through DMAIC, can be adapted to the context of Emergency Care Units, thereby promoting data-driven governance and organizational learning. It also reinforces the importance of consolidating visual routines, indicators, and continuous feedback cycles to sustain improvements in complex, resource-limited environments—a typical condition in SUS. Finally, by proposing a set of indicators integrated into both operational and documentation management, the study contributes to the development of a replicable model of biosafety, efficiency, and professional engagement, aligned with the guidelines of RDC and the institutional sustainability principles highlighted [16,18].

This study is subject to several limitations. First, the analysis was conducted across only three ECUs, limiting the empirical breadth of the findings. Although this design aligns with the logic of analytical generalization used in multiple-case studies, the results should not be interpreted as statistically generalizable. The small number of units reflects operational constraints typical of health-service research involving direct observation, analysis of internal routines, and verification of institutional documentation.

Second, the study focused on diagnostic assessment and the development of an improvement plan, without empirically implementing the proposed actions. While consistent with the research’s exploratory purpose, this limits the ability to conclude the effectiveness of the improvement interventions. Future studies could adopt a longitudinal approach to evaluate performance gains, safety outcomes, and behavioral adherence following the practical application of the DMAIC.

Additionally, the data collection process relied on direct observation and interaction with operational teams, which may be influenced by researcher presence and local supervisory dynamics. Although reliability was enhanced by having all observations conducted by the same trained researcher, independent evaluators or digital traceability tools could further strengthen methodological rigor.

Despite these limitations, the study provides valuable evidence on managerial, behavioral, and documentation-related factors influencing sharps waste management in resource-constrained healthcare settings, thereby contributing to improvements in governance, occupational safety, and regulatory compliance.

## 5. Conclusions

The LSS approach was adopted in this study as an analytical and planning framework to examine fragmented operational practices and to structure standardized, monitorable, and sustainable process proposals aligned with RDC requirements and institutional biosafety principles. The diagnostic results indicated that the leading causes of non-conformity were predominantly managerial and behavioral factors, including the absence of formal routines, labeling failures, training gaps, and weak documentation practices. The use of the DMAIC cycle enabled the identification of these causes and supported the development of practical and low-cost proposed solutions, such as visual checklists, standardized routes, performance indicators (KPIs), and managerial feedback routines. These proposed measures are expected to reduce process variability, strengthen waste traceability, and foster a culture of shared responsibility among managers and operational teams.

Beyond technical and regulatory considerations, the proposed improvements are expected to influence organizational climate and professional motivation. Safer and more predictable environments, if achieved through future implementation, may reduce anxiety about accidents and contamination, bolster confidence in institutional routines, and encourage engagement in biosafety practices. Such effects are discussed in the literature as being associated with productivity and staff retention, highlighting the importance of managerial strategies that integrate safety, learning, and well-being in the workplace. The findings also suggest that sharps waste management transcends technical aspects and constitutes a continuous educational and psychological process. The proposed introduction of visual routines, feedback audits, and short training sessions is described as having the potential to function as mechanisms for on-the-job learning, fostering preventive behaviors, and strengthening biosafety culture. Within this scope, DMAIC is positioned as a management and educational framework, rather than evidence of implemented outcomes.

As a practical contribution, the analytical framework and action plan proposed in this study may be replicated in other public health units, subject to contextual adaptation, demonstrating that LSS tools have the potential to support efficiency and governance even under budget constraints. From a scientific perspective, the study contributes to expanding the understanding of DMAIC applicability in public services, reinforcing its potential as an instrument for integrating biosafety, process management, and institutional sustainability. Strengthening sharps waste management is discussed here as relying not only on structural investments but also on the consolidation of data-driven managerial practices, supported by indicators and a culture of continuous learning—key pillars identified in the literature for occupational safety, regulatory compliance, and healthcare sustainability.

Beyond operational and biosafety implications, the diagnostic findings support discussion of how routine standardization, reduced variability, and stronger documentation governance may generate economic benefits for emergency care units, particularly in budget-constrained environments. Although these projections were not empirically tested, they represent a promising avenue for future longitudinal research to assess the financial implications of implementing DMAIC-based improvement initiatives in public healthcare settings.

As a future research agenda, we recommend empirically implementing and longitudinally evaluating the proposed improvement plan to assess its effects on process variability, occupational safety, documentation compliance, and resource efficiency. Longitudinal studies would allow a more robust assessment of operational, behavioral, and economic outcomes associated with DMAIC application. Expanded investigations across healthcare units with different levels of complexity and governance maturity may further validate and refine the analytical contributions of this research.

## Figures and Tables

**Table 1:** Cause–Effect Matrix for Sharps-Waste Processes (5M).

Axis (5M)	Empirical Evidence	Impact on CTQ
Method	Absence of a fixed route and fill-level verification; non-standardized replacement criterion.	Process variability; overflow risk.
Manpower	Sporadic, unsystematic training.	Poor knowledge of legal requirements; handling errors; non-conformities.
Material	Missing labels/pictograms.	Biosafety violations; sanitary liability.
Measurement	No performance indicators in place.	Impossible to establish baselines and targets.
Milieu (Environment)	Lack of proof of final disposal.	Environmental risk and legal liability.

**Table 2:** GUT Prioritization Summary.

Cause (5M Category)	Gravity (1–5)	Urgency (1–5)	Trend (1–5)	Total Score
Absence of standardized verification routines (Method)	5	5	4	14
Lack of systematic and documented staff training (Manpower)	5	4	4	13
Incomplete documentation of final disposal certificates (Milieu)	4	4	4	12
Missing labeling/pictograms (Material)	4	3	3	10
Absence of monitoring indicators (Measurement)	3	3	3	9

**Table 3:** RDC Compliance Checklist and Site-Level Summary.

RDC Item	Field Finding	Compliance
Rigid container with proper identification (“Sharps waste”, Group E)	Containers are sufficiently rigid; however, identification is irregular at some points.	Partial
Label/pictogram at collection points	Generic label; absence of the specific biosafety pictogram in several rooms.	No
Continuing education program (Art. 91)	Occasional training; no systematic routine or records.	No
PGRSS is available to workers	The document exists, but it is not accessible to the ECUs.	No
Internal transport with a defined route	Fixed schedule, but no standardized route across shifts.	Partial

**Table 4:** Continuous Improvement Plan: Actions, Tools, and Deadlines.

CTQ	Proposed Improvement Action	LSS Tool	Target Deadline
100% documentary conformity	Implement a 5S checklist and poka-yoke for labeling and identification (per ANVISA standards).	Kaizen Blitz	3 months
0 sharps incidents/year	Quarterly training with simulation focused on handling, segregation, and PPE.	DMAIC (Improve)	6 months
Standardized internal route (σ < 1 min)	Value Stream Mapping (VSM) and spaghetti chart and route/layout redesign.	VSM	4 months
100% box replacement at ¾ capacity	Visual Kanban cards on collectors to trigger exchange.	Kanban	2 months
100% evidence of final disposal	Digital traceability module attached to the PGRSS for certificate archiving.	SIPOC + CTQ flow-down	6 months

**Table 5:** Proposed KPI Dictionary for the Control Phase.

KPI	Definition/Measurement	Target	Data Source & Review Cadence
% of containers with correct labeling	Share of inspected containers that meet ANVISA labeling and biosafety pictogram requirements.	100%	Field checklist/poka-yoke verification; monthly review during gemba.
Sharps-incident rate per 100 employees-month	The number of sharps accidents is normalized by 100 employees-month to avoid headcount distortion.	0 incidents/year (targeting zero harm)	Incident forms and occupational-safety logs; monthly trend check and semiannual audit.
Internal-flow lead time (minutes)	Time from point of generation to temporary storage; tracked for mean and variability (σ).	Low and stable σ (e.g., σ < 1 min) with downward mean trend	VSM timing sheets/route observations; monthly gemba; layout revisited if out of control.
% of disposal certificates archived on time	Share of final-disposal certificates archived within the required timeframe (per contract/regulation).	100%	Digital PGRSS module/records office; continuous dashboard + semiannual regulatory audit.

## Data Availability

Dataset available on request from the authors.

## Appendix A. Checklist Criteria Derived from ANVISA Resolution RDC No. 222/2018

**Table A1:** Checklist Criteria applied in observation.

Domain	Criterion/Requirement	Evaluation Method
Infrastructure	1. Presence of rigid, puncture-resistant containers labeled “Sharps Waste—Group E.”	Direct observation
	2. Containers positioned near points of waste generation.	Field inspection
	3. Adequate temporary storage room (ventilated, illuminated, restricted access).	Field inspection
	4. Proper signage and biosafety symbols at collection and storage points.	Field inspection
	5. Evidence of cleaning and maintenance of containers and collection carts.	Documentation/observation
Process Flow	6. Existence of a defined internal transport route for sharps waste.	Interview/field observation
	7. Fixed schedule for internal collection.	Document/time record review
	8. Container replacement criterion based on ¾ fill-level or 24 h.	Observation checklist
	9. Use of personal protective equipment (PPE) during collection.	Direct observation
	10. Visual verification or checklist routine for container status and labeling.	Observation/record review
Documentation Governance	11. Availability of the Healthcare Waste Management Plan (PGRSS) on site.	Document verification
	12. Evidence of staff training in biosafety and waste management.	Training record review
	13. Availability of recent training attendance lists.	Record review
	14. Archive of final disposal certificates issued by the licensed contractor.	Document verification
	15. Periodic review and updating of the PGRSS documentation.	Interview/record review

## Appendix B. Qualitative Field Observations Supporting the 5M Cause–Effect Matrix

**Table A2:** 5M observed in the cases evaluated.

ECUs (Municipality, State)	5M Axis	Observed Practice	Evidence Source	Related Non-Conformity/Risk
ECU São José (Imperatriz–MA)	Method	Absence of a fixed internal transport route and inconsistent verification of container fill levels	Direct on-site observation; RDC checklist	Process variability; risk of overflow
	Manpower	Training activities are conducted sporadically and without standardized content.	Field verification; brief staff interaction	Knowledge gaps; handling errors
	Material	Sharps containers without labels or pictograms in some areas	Direct observation	Biosafety non-compliance
	Measurement	No indicators defined for monitoring waste generation or replacement frequency	Document review	Inability to establish baselines and targets
	Milieu (Environment)	Lack of documented proof of final disposal available on-site	Document review	Environmental and legal risk
ECU Augustinópolis (TO)	Method	Non-standardized criteria for container replacement and route definition	Direct observation; RDC checklist	Process variability
	Manpower	Inconsistent training practices across shifts	Field verification	Risk of non-conformities
	Material	Missing identification labels on waste containers	Direct observation	Biosafety violations
	Measurement	Absence of performance indicators related to waste handling	Document review	Lack of monitoring capability
	Milieu (Environment)	Final disposal records are not readily accessible at the unit	Document review	Environmental liability
ECU Cidade Bom Jardim (Parauapebas–PA)	Method	Internal transport routines vary by shift	Direct observation	Process variability
	Manpower	Limited staff awareness of regulatory requirements	Brief staff interaction	Handling errors
	Material	Incomplete visual identification of containers	Direct observation	Biosafety risk
	Measurement	No formal metrics for control of sharps waste flow	Document review	Lack of baselines
	Milieu (Environment)	Absence of on-site evidence confirming final disposal	Document review	Environmental and legal risk

## Appendix C. Site-Level GUT Scoring Supporting the Prioritization Summary

**Table A3:** GUT’s summary of the evaluated cases.

Cause (5M Category)	SJ-G	SJ-U	SJ-T	AU-G	AU-U	AU-T	PA-G	PA-U	PA-T	Mean G	Mean U	Mean T	Consolidated Score
Absence of standardized verification routines (Method)	5	5	4	5	4	4	5	5	4	5.0	4.7	4.0	14
Lack of systematic staff training (Manpower)	5	4	4	5	4	3	5	4	5	5.0	4.0	4.0	13
Incomplete disposal documentation (Milieu)	4	4	4	4	3	4	4	5	4	4.0	4.0	4.0	12
Missing labeling/pictograms (Material)	4	3	3	4	3	2	4	4	4	4.0	3.3	3.0	10
Absence of monitoring indicators (Measurement)	3	3	3	3	2	3	3	4	3	3.0	3.0	3.0	9

Legend: SJ = ECU São José (Imperatriz–MA); AU = ECU Augustinópolis (TO); PA = ECU Bom Jardim (Parauapebas–PA); G = Gravity; U = Urgency; T = Trend; Scores range from 1 (lowest) to 5 (highest).

## Appendix D. Disaggregated RDC Compliance Evidence by Emergency Care Unit

**Table A4:** RDC per emergency care unit.

RDC Item	Indicator (Operational Criterion)	São José (MA)	Augustinópolis (TO)	Bom Jardim (PA)
Rigid container with proper identification (Group E)	% of containers correctly labeled	78%	65%	82%
	Containers meeting the puncture-resistance requirement	Yes	Yes	Yes
Label/pictogram at collection points	% of rooms with biosafety pictogram	40%	25%	55%
Continuing education program	Formal training sessions/year	1	0	1
	Existence of training records	No	No	Partial
PGRSS available to workers	PGRSS is physically accessible on-site	No	No	Yes
	Staff aware of PGRSS location (%)	20%	15%	45%
Internal transport with a defined route	Existence of a mapped internal route	Partial	No	Partial
	Route consistency across shifts (%)	60%	35%	70%
